# Pathophysiological effects of Tamiflu on liver and kidneys of male rats

**DOI:** 10.1186/s43088-021-00189-6

**Published:** 2022-01-25

**Authors:** Wafaa H. Abdel-Ghaffar, Eman A. Abdelghffar

**Affiliations:** 1 Department of Zoology, Faculty of Science, Ain Shams University, Cairo, Egypt; 2 Biology Department, Faculty of Science, Taibah University, Yanbu Branch, Saudi Arabia

**Keywords:** Histopathology, Biochemical analyses, DNA electrophoresis, Oseltamivir phosphate, Rats

## Abstract

**Background:**

Tamiflu/oseltamivir phosphate (OP), an anti-influenza drug, has a highly doubted safety especially after many cases of abnormal behaviour and deaths reported after being used. Such controversy was also locally and globally generated, especially after being heavily used in COVID-19 treatment protocol. This study was designed to evaluate the effect of three different doses of OP on the liver and kidneys of male adult albino rats through histological approaches, measuring their DNA integrity and biochemical analyses. Different doses of Tamiflu applied to humans were converted to rats, then observed their effects on the liver and kidneys. Rats were divided into four groups. G1: considered as control group. The rest of the three treated groups were received the same calculated dose of Tamiflu (6.75 mg/kg b.w.) in three different durations. G2, G3 and G4 represented the animals orally received OP, in which the rats received OP twice for 5 consecutive days, once for 10 and 45 days, respectively.

**Results:**

Our data showed numerous deleterious necrotic and fibrotic histopathological changes in the liver, and kidneys; as well as necrotic DNA smears, by using electrophoresis, in OP-treated rats of G2 and G4. In addition, OP significantly increased the serum cellular hepatic/renal toxicity markers (ALT, AST, ALP, GGT, indirect bilirubin, urea, creatinine, uric acid, & Na^+^). Also, it showed a reduction in the levels of serum total protein, albumin and K^+^ ions in rats of G2 and G4 compared with G1. In G3, OP treatment did not significantly alter hepatic/renal histological, DNA integrity and biochemical analyses in rats.

**Conclusions:**

The therapeutic and long-term prophylactic doses of OP most likely cause structural and functional hepato- and nephrotoxicity in experimentally subjected rats. So, caution must be taken during Tamiflu treatment, and not used for long durations and/or with repetitive doses (time- and/or accumulative-dose-dependent); especially with patients suffer from liver and/or kidney dysfunction, while the short-term prophylactic dose of OP appears to be relatively safe and could be explored for oral medications.

**Graphical Abstract:**

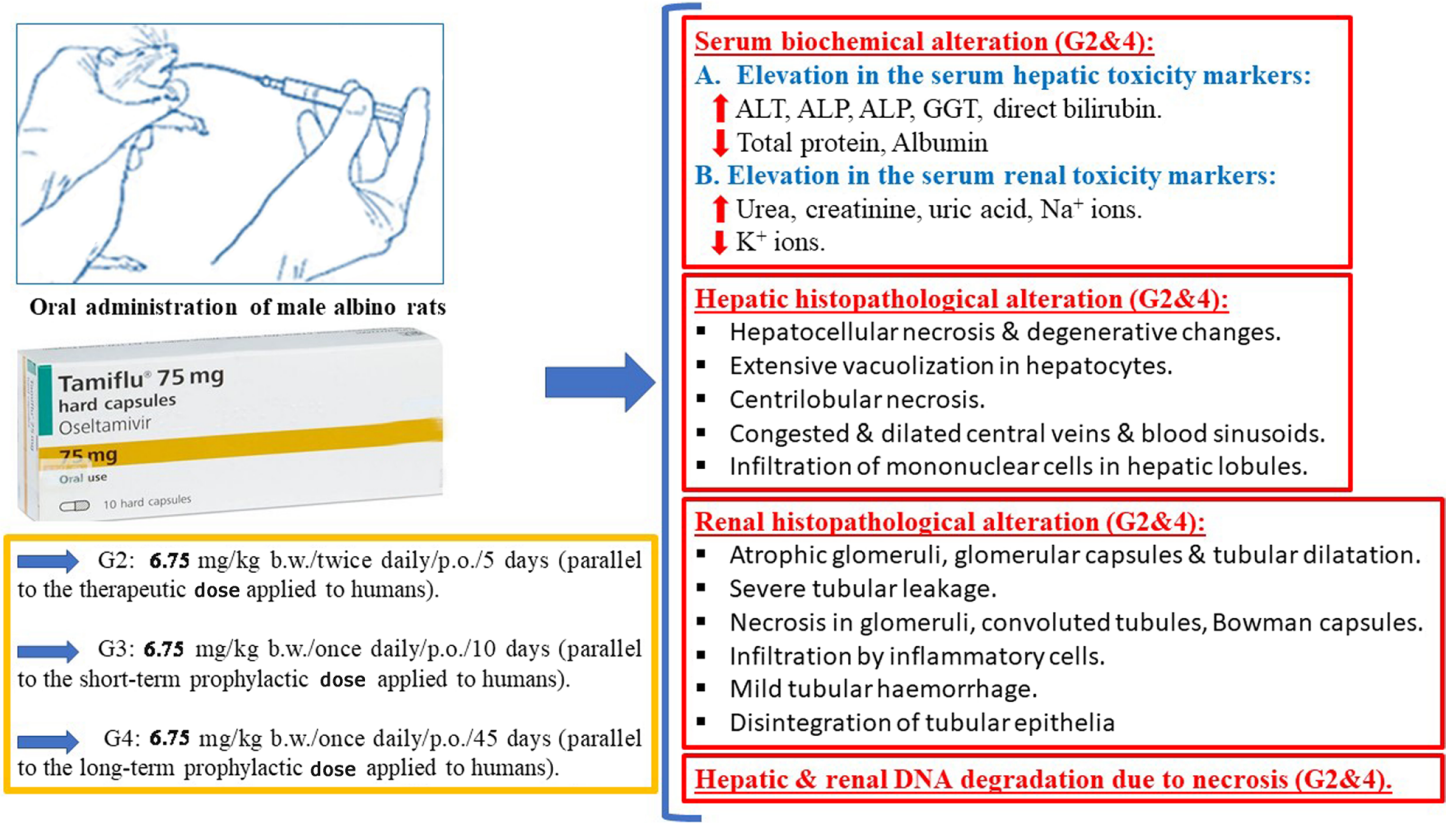

## Background

Recently, Tamiflu (oseltamivir phosphate, OP)—working as neuraminidase inhibitor—received high attention because of COVID-19 pandemic. In 1998, Mendel et al. [1] stated that Tamiflu is designed to be highly specific to the influenza virus. Due to this high specificity, it was suggested that Tamiflu would be effective in the treatment of the coronavirus. But, unfortunately it is worth to mention that the laboratory testing—conducted by Hong Kong University—demonstrated that OP did not have any antiviral effect on the novel coronavirus [2]. Also, the effectiveness of Tamiflu is doubted among researchers, due to publication bias policy (publishing favourable studies and not publishing unfavourable studies) adapted by its original manufacturer—Roche—that refused to release its own trial data [3]. So, it would not be possible to know the actual side effects until all Roche's data are being honestly published [4–6]. Tamiflu is indicated as prophylactic influenza drug and as therapeutic for uncomplicated acute illness due to influenza in patients 1-year-old and the older ones who have been symptomatic for no more than 2 days [7]. However, the efficacy and efficiency of Tamiflu treatment (reduction of the viral serious complications and hospitalization) is still subject of debate. Several authors studied the safety, and tolerability of OP—since the early beginning of drug launching—[8, 9], and recommended its usage. Conversely, others reported that Tamiflu administration cause several adverse effects on many vital organs [10–14].

In fact, from another critical point of view, Tamiflu was tested in 1998 and released as a drug in 2001. According to FDA, the newly developed drug requires several tests designed (in vitro**/**in vivo) to determine its major toxicities prior to be first used by man [15]. FDA (1991) [16] published that the drugs examination protocols and the procedures take an average of 10 years for an experimental drug to travel from the laboratory to the public pharmacies in USA—which is inconsistent with what was happened with Tamiflu (FDA four main phases: the average period per each phase differ, and ranged from 1–3.5 years). The latter statements oppose the fact that Tamiflu is being licensed after two years only! Although Tamiflu is already manufactured in USA, these rules did not apply to it!

So, conflicts between supports and opponents emerged (because of Tamiflu pathological alterations’) to use Tamiflu as a therapeutic and prophylactic drug that is coincided with the lack of fixed; unified, complete, clear or even justified data bases. Collectively, from all of the aforementioned reasons, this designed study became a must. This study designed to evaluate the effect of three different doses of Tamiflu itself as a drug on the main vital organs represented by the liver and kidneys. The resulted drug alterations will be assessed histopathologically, biochemically, in addition to DNA electrophoretic analyses.

## Methods

### Chemicals and drug

TAMIFLU®/oseltamivir phosphate (75 mg) is manufactured by F. Hoffman-La Roche, Gilead Sciences, Foster City, California, USA. It is available in the market as capsules for oral administration. ALT (Alanine aminotransferase), AST (aspartate aminotransferase), alkaline phosphatase (ALP), gamma-glutamyltransferase (GGT), total and direct bilirubin, total protein, albumin, urea, creatinine, uric acid, sodium (Na^+^) and potassium (K^+^) were colorimetrically determined by using Bio-diagnostic Kits (Giza, Egypt). All of the other used chemicals, solvents and reagents were of analytical and pure grade. Also, indirect bilirubin was calculated as total bilirubin value minus direct bilirubin value.

### Animals

Forty male adult Wistar albino rats (Rattus norvegicus), ranging between 3 and 4 months and weighing 150–200 g, were obtained from the Institution of Animal Veterinary and Serum Vaccine Research, Abbassia (Cairo, Egypt). The rats have been housed in suitable-sized cages in the animal house of Zoology Department, Faculty of Science, Ain Shams University, for 2 weeks as an accommodation period for them. Water and standard pellets of animal diet were supplied ad libitum.

### Experimental design and treatment schedule calculation:

The rats were randomly divided into 4 groups (*n* = 10) as follows: Group 1 (G1): The control animals, which received (orally/daily) distilled water. All of the treated groups (G2–G4) were orally administrated by 6.75 mg/kg daily, but differ in the duration as follows: Group 2 (G2): The animals received Tamiflu twice, for 5 consecutive days according to Lee et al*.* [17]. This treatment is parallel to the therapeutic dose applied to humans (75 mg/70 kg b. w. of Tamiflu twice orally/daily for 5 consecutive days). Group 3 (G3): The rats received Tamiflu once for 10 days according to Hayden et al*.* [18]. This treatment is parallel to the short-term prophylactic dose applied to humans (75 mg/70 kg b. w. of Tamiflu orally/daily for 10 consecutive days). Group 4 (G4): The animals received Tamiflu once for 45 days according to Peter et al*.* [19]. This treatment is parallel to the long-term prophylactic dose applied to humans. The above dosages were converted to rats according to the equation “Table” given by Paget and Barnes [20], as follows:

$$\begin{gathered} {\text{Animal}}\;{\text{dose }}({\text{mg}}/{2}00\;{\text{g}}) \, = {\text{ Dose}}\;{\text{of}}\;{\text{the}}\;{\text{humanweighing}}\;{7}0\;{\text{kg}} \times {\text{conversion}}\;{\text{factor}} \hfill \\ {\text{Animal}}\;{\text{dose }}({\text{mg}}/{2}00\;{\text{g}}) \, = {75} \times 0.0{18} = {1}.{35}\;{\text{mg}}/{2}00\;{\text{g}} \hfill \\ {\text{Animal}}\;{\text{dose }}({\text{mg}}/{\text{kg}}) \, = { 1}.{35} \times {5 } = {6}.{75}\;{\text{mg}}/{\text{kg}} \hfill \\ \end{gathered}$$

To prepare a solution of 75 mg of the drug, the powder inside the gelatinous capsule (75 mg of Tamiflu) was dissolved in 75 ml distilled water. So, each 1 mg of the drug will be dissolved in 1 ml of the prepared solution. The prepared drug was administrated orally by stomach tube that contained the calculated dose applied orally to rats in different durations according to the group.

### Blood collection and biochemical investigations

The blood samples were immediately collected in plain test tubes after scarifying the anaesthetized rats, and left to clot, then centrifuged (5000 rpm, 15 min). The sera were kept at -80 °C for further biochemical analyses (ALT, AST, ALP, GGT, bilirubin profiles, protein, albumin, urea, creatinine, uric acid, electrolytes as Na^+^ and K^+^).

### Tissues sampling and histological investigations

The dissected liver and the kidneys were cut into small pieces (≈0.5 cm). The pieces were washed in saline, fixed in liquid Bouin’s solution, then processed and stained in H&E for histopathology. Sections were photographed using a digital microscope camera (Hirocam, MA88-500 Eyepiece) attached to a Magnus microscope (MLX-B Plus SP, China). For each renal section, 3 random non-overlapping high power areas were graded semi-quantitatively for lesion severity according to the following scoring system: 0, no lesion; 1, mild damage; 2, moderate damage; 3, severe damage.

### Molecular investigations: DNA fragmentation

For DNA extraction and fragmentation assay (apoptosis or necrosis detection), the used method based on salting out extraction (refer to Aljanabi and Martinez [21], and modified by HassabEl-Nabi [22]). In this method, 10 mg liver or kidneys’ tissues was squeezed in micro tubes and lysed by using 600 µl lysing solution (50 mM NaCl, 10 mM Tris, 10 mM Na_2_ EDTA, 0.5% SDS, pH 8.3), and then shaken gently. Finally, after several technical steps, the resulted mixture of each tube was directly loaded on agarose gel. The gels were photographed using a digital camera; the DNA was visualized as necrotic smears which were measured using Gel-Pro program.

### Statistics

Data were presented as mean ± SE. Statistical analysis was performed with one-way ANOVA for biochemical and electrophoretic analyses of this study using Graph Pad Prism software (version 5.3 Bic Executable, Graph Pad Software Inc.). The optical density of the DNA necrotic damage was detected by using Image G program. *P* value of < 0.05 was considered significant.

## Results

### Histological findings

#### The liver

**Fig. 1:**
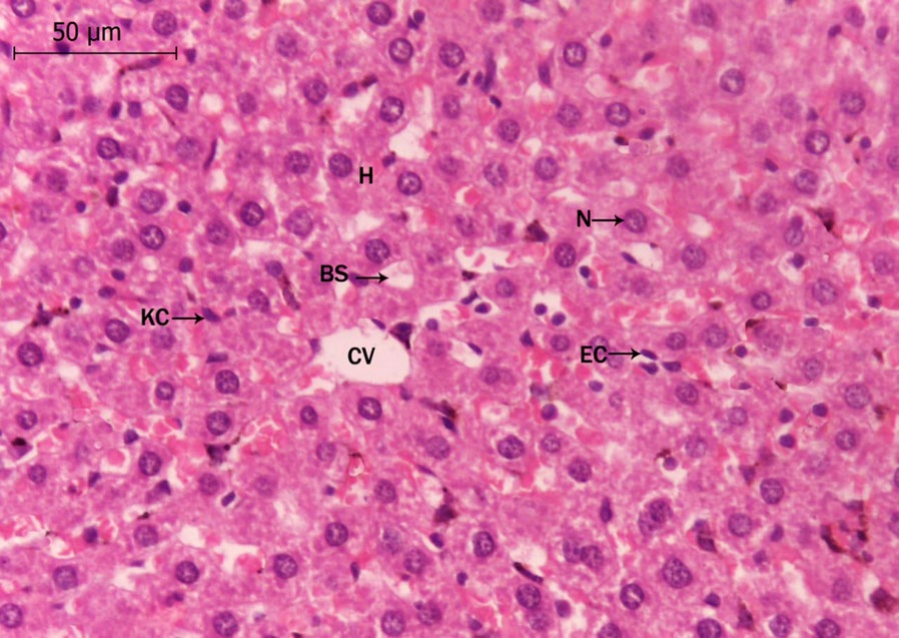
A photomicrograph of a control liver section of a rat showing the typical structure of the liver cells (H), forming a network around the central vein (CV). Notice that the liver cells’ nuclei (N) are large, spherical and almost centrally located. Notice Küpffer cells (KC) in association with the blood sinusoids (BS), and endothelial cells (EC, X 400, H&E)

**Fig. 2:**
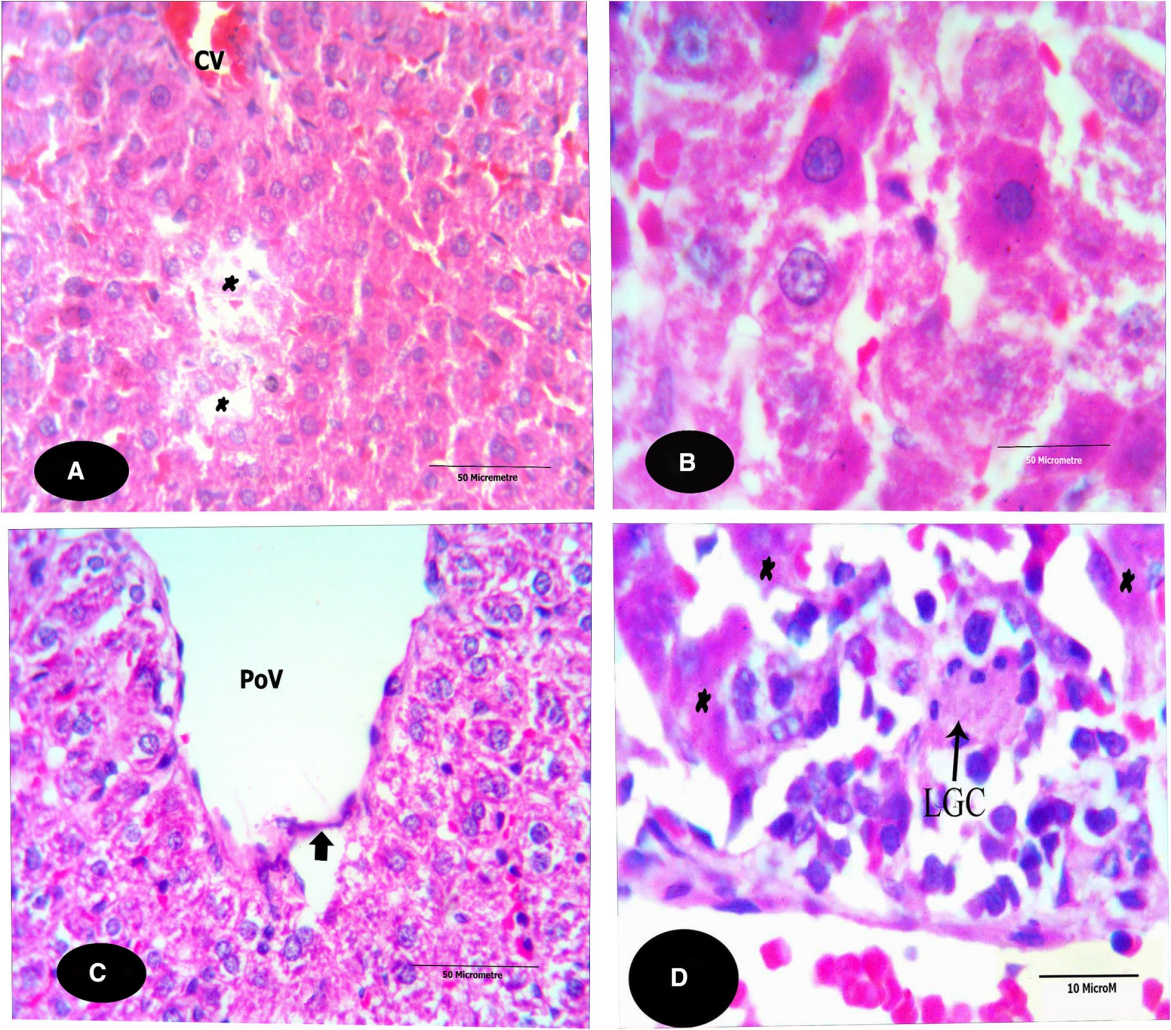
**A–D** Photomicrographs of liver sections of G2 stained with H&E. **A** shows areas of faintly stained autolysed hepatocytes (*). The blood sinusoids and the CV are congested with blood. (X100). **B** displays swollen and basophilic-stained hepatocytes. The hepatocytes exhibit pleomorphic nuclei. The nuclei are either pyknotic or vanished. (X1000). **C** shows a dilated branch of the portal vein (PoV) which exhibits a deformed and ruptured endothelial lining. The tissue architecture is disrupted. Notice the autolysed narrow area of the tissue underneath the endothelial lining of the vein. The cytoplasm of the hepatocytes is coagulated and vacuolated. Also, the hepatocytes’ nuclei are shrunken. (X100). **D** shows an enlarged area nearby a CV (at the bottom of the figure). The hepatocytes show coagulative necrosis (*). Also, a Langhans giant cell (LGC) invades the area; it presumably scavenges the degenerated cells. (X1000)

**Fig. 3:**
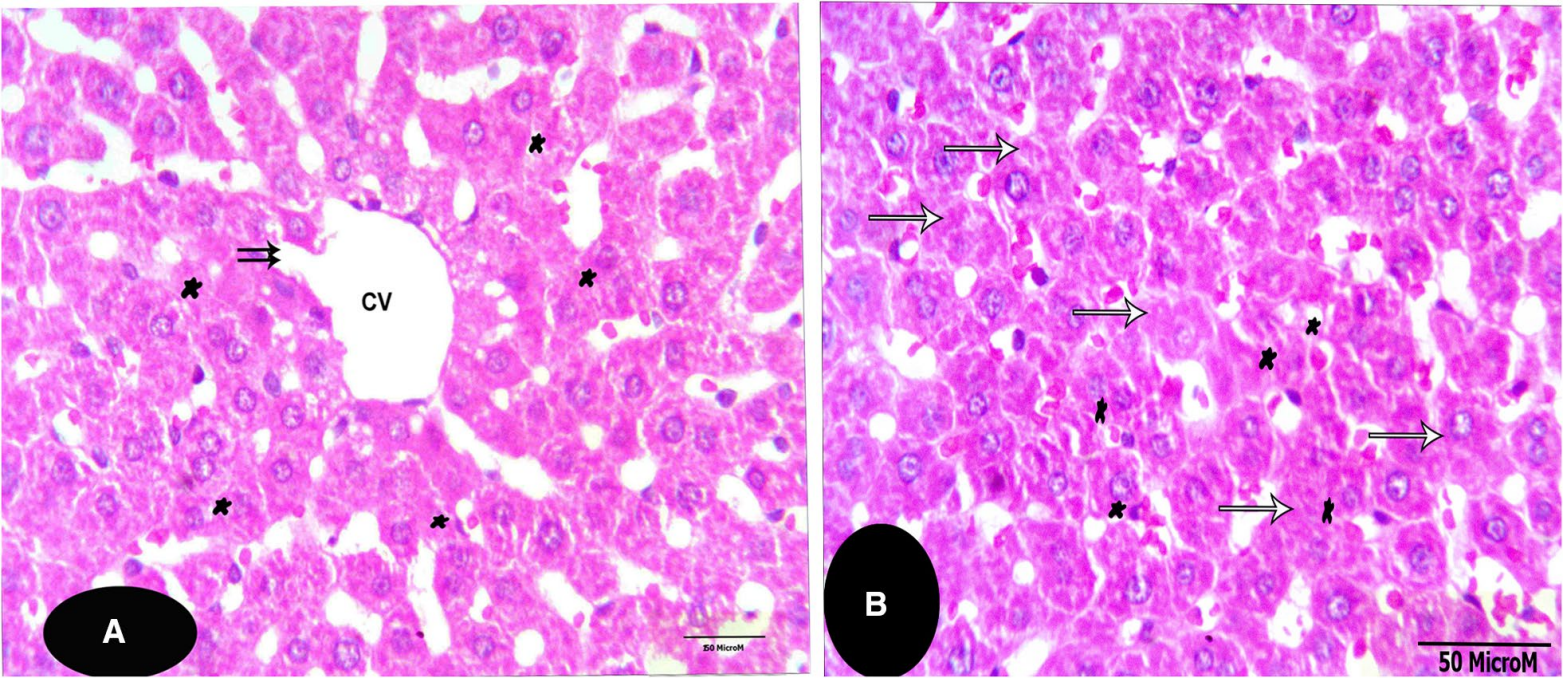
**A**,** B** Photomicrographs of liver sections of G3 (X 400, H&E). **A** displays a central vein (CV) with a partially eroded wall (arrows). Some hepatocytes display foamy cytoplasm (*), while the rest of hepatocytes are shrunken, and exhibit darkly stained cytoplasm. **B** shows hepatocytes with foamy cytoplasm (*). The hepatocytes exhibit degenerated and karyolysed nuclei (arrows). The nuclei of other hepatocytes display abnormal chromatin distribution

**Fig. 4:**
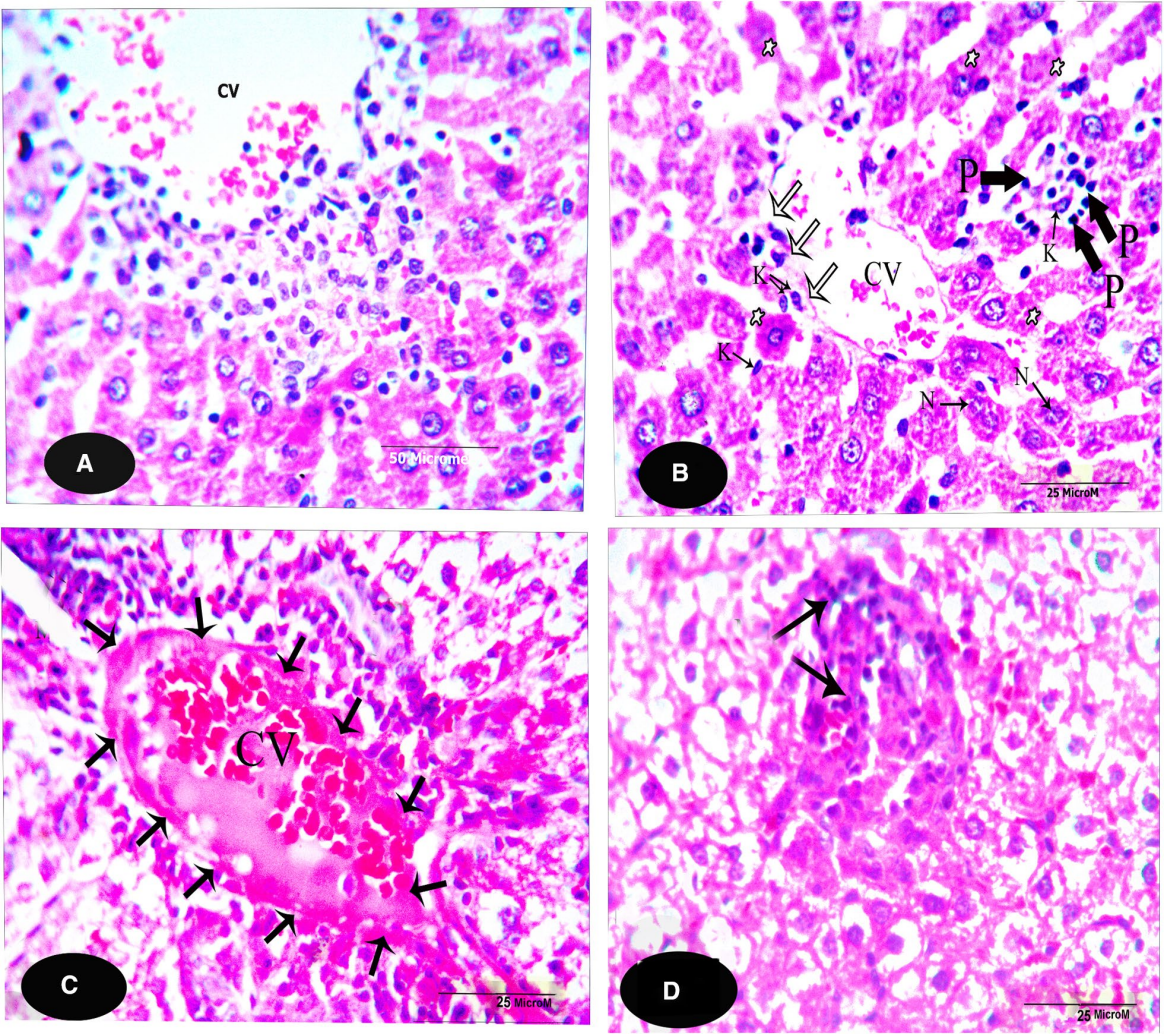
**A–D** Photomicrographs of a liver sections of **G4** (X 400, H&E). **A** focuses on the eroded CV which is accompanied with heavy accumulation of mononucleated cells. Generally, the hepatocytes’ nuclei in such necrotic area exhibit abnormal chromatin distribution. **B** shows a CV that displays eroded endothelium lining (white arrows). The mononuclear cells invade the eroded area around the central vein. Generally, most of the hepatocytes are shrunken, degenerated and darkly stained; this is accompanied with widening of the BS. Notice that the hepatocytes’ nuclei are pleomorphic. **C** shows a dilated CV that displays a marked thickening of its endothelial lining cells (arrows). The central vein’s lumen is largely occluded with a mass of blood clot which, apart from a few remains of red blood cells, is homogeneous, hyaline and acidophilic. **D** shows a necrotic area of hepatocytes (arrows). Most of the hepatocytes are highly evacuated (vanished cytoplasm)

The microscopical examination of the control liver sections (Fig. 1) reveals normal structure of hepatocytes. The examination of the liver sections of Tamiflu-treated groups (G2 to G4) showed variable histopathological alterations. In G2: the examination of this group revealed from mild to moderate alterations. Mostly, the blood sinusoids together with the central veins were congested (Fig. 2A). Also, the hepatocytes were either swollen and showed basophilic-stainability or shrunken and vanished (Fig. 2A, B). In addition, most branches of the hepatic portal veins were dilated with a deformed and ruptured endothelial lining as shown in Fig. 2C. The hepatocytes’ were either necrotic, pyknotis, pleomorphic or even vanished. The cytoplasm of the hepatocytes showed coagulation and vacuolation. Numerous inflammatory cells **as** Langhans giant cells invaded the deteriorated areas of the liver sections; it presumably scavenges the degenerated cells (as shown in Fig. 2D). In G3: almost normal hepatocytes were shown except few affected spots (Fig. 3A, B). These spots exhibited degenerated hepatocytes with darkly stained cytoplasm located nearby the eroded endothelium of the affected central veins (Fig. 3A). Most hepatocytes exhibited foamy cytoplasm (Fig. 3A, B). Non-homogeneous and sever picture of alterations was observed in group 4. The examination of the liver sections of G4 showed focal coagulative necrosis (Fig. 4A, B) of hepatocytes that surrounded the central vein. This is accompanied with heavy accumulation of mononucleated cells that invaded the destructed area. The nuclei showed abnormal chromatin distribution. By dosage accumulation and long period of oral administration, the hepatocytes showed areas of vacuolar and vanished cytoplasm (Fig. 4C, D).

#### The kidneys

**Fig. 5:**
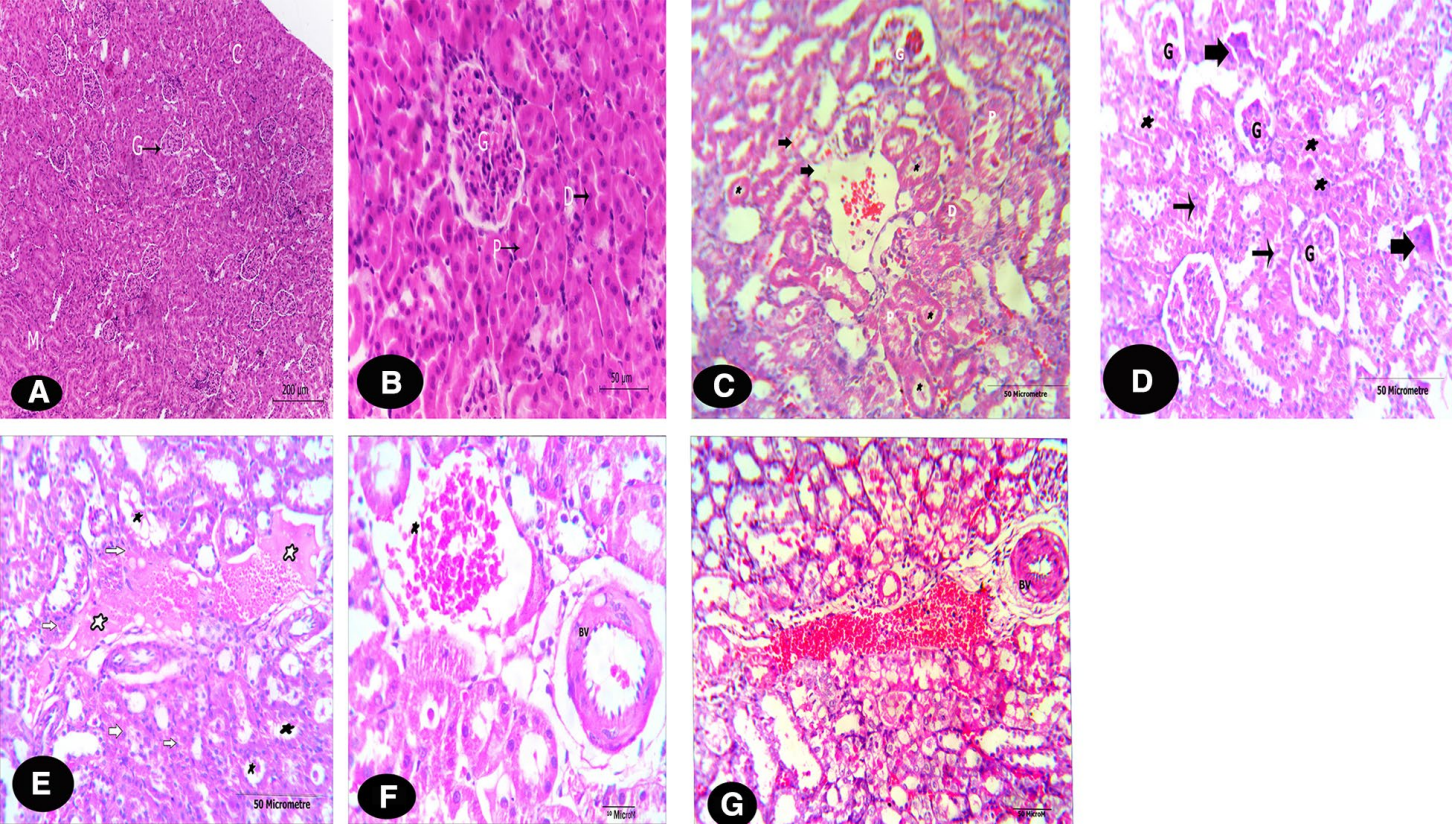
**A–G** Photomicrographs of T. S. of kidneys of rats of G1 showing part of the cortices (C, H&E). **A** The cortex is characterized by the presence of numerous glomeruli (G). (X 100, H&E). **B** A magnified part of the cortex of **A** exhibit normal typical structure of G, proximal convoluted tubules (P) and distal convoluted tubules (D). (X 400, H&E)**. C** A photomicrograph of T. S. of kidney of G2 showing proximal (P) & distal (D) tubular necrosis with variable degrees of stainability. The necrotic cells fell into the tubules’ lumen and obliterated them (*). Notice the vacuolar changes (arrows), the G degeneration and capsular thickening. (X 100, **G2,** H&E). **D** A photomicrograph of T. S. of kidney of G3 showing moderate alterations restricted to few spots per each examined section, and hypercellularity (*). The glomerular alterations include clefted and shrunken glomeruli (G and arrow, respectively). (X 100, **G3,** H&E). **E** A photomicrographs of T. S. of kidneys of G4 showing sever degradation of the kidney tubules (white-arrows). In most cases, the tubules show obliterated lumina (black asterisk), and hypercellularity in between. The interstitial areas among the renal tubules together with the lumina of the renal tubules were congested with blood cells (white asterisk). Notice the cellular infiltration (thin black arrow). (X 100, **G4**). **F** & **G** showing degenerated, widened, and thickened BVs, plus severe vacuolar degeneration. Notice variable degrees of necrotic changes in both sections. The interstitial areas among the renal tubules together with the lumina of the renal tubules were congested with blood cells. (X 400 and 100; respectively, **G4**)

**Fig. 6:**
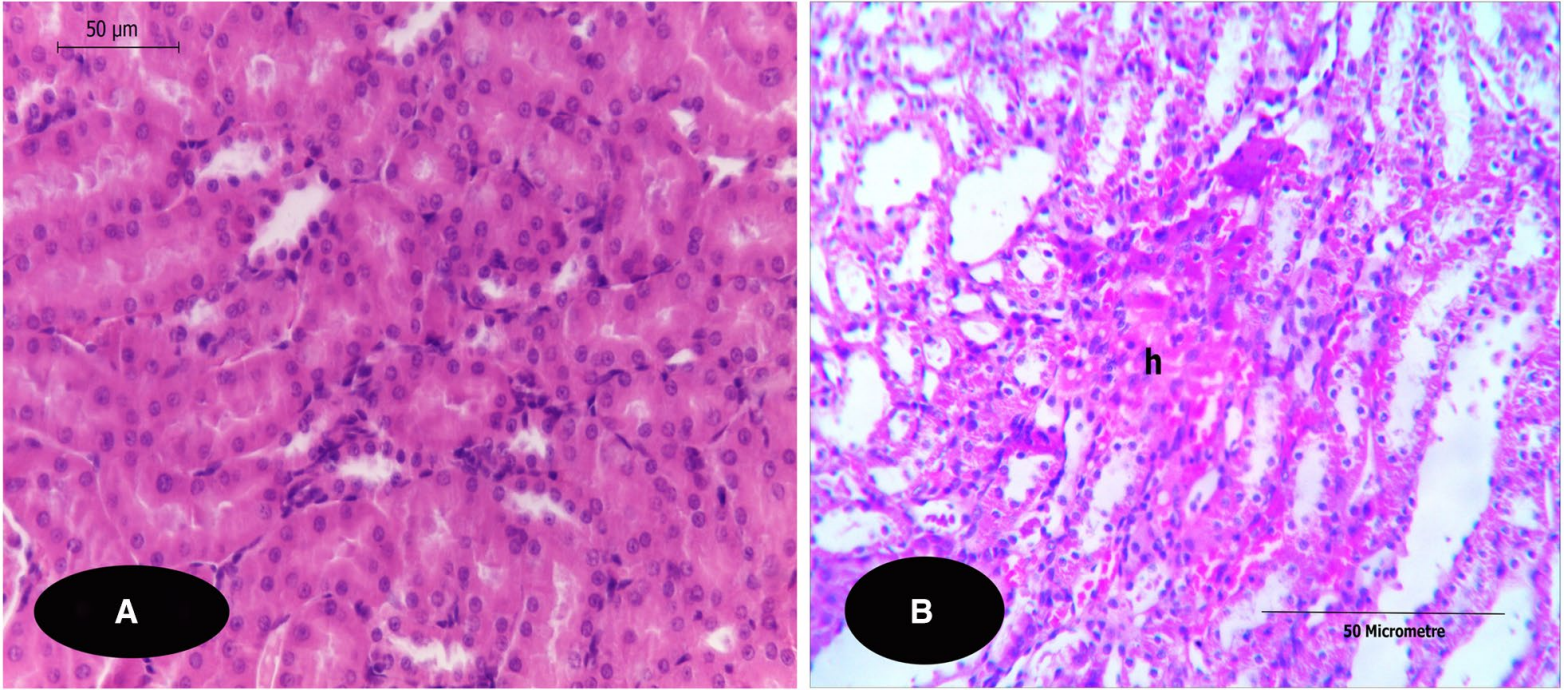
**A**,** B** T. S. of kidneys of rats show parts of the medullae stained with H&E. **A** T. S. of a kidney of G1 (control rat) showing a typical structures of the medullary tubules. (X 400). **B** T. S. of a kidney of **G4** showing blood congestion/haemorrhage (h) which is detected on the medullary region in of this group (X 100). Notice: both **G2&3** did not show any histopathological alterations in the medullary regions of the examined kidney sections compared to **G1** sections

The microscopical examination of the control rats’ kidneys shows typical normal structure of the cortex and medulla (Fig. 5A, B & Fig. 6A and Table 1). The histological sections of the kidneys of Tamiflu-treated groups (G2, G3 and G4) are presented by Figs. 5C–F & 6B.

**Table 1:** The effect of Tamiflu on renal histology of various treated groups compared to that of the control group

	G1	G2	G3	G4
*Cortices alterations*				
Glomerulus changes	0.00 ± 0.00	1.38 ± 0.23^***^	0.33 ± 0.80	2.48 ± 0.20^***^
Capsular thickening	0.00 ± 0.00	0.91 ± 0.26^**^	0.19 ± 0.03	1.12 ± 0.18^***^
Proximal con. tubule necrosis	0.00 ± 0.00	1.80 ± 0.07^***^	0.36 ± 0.10	2.65 ± 0.14^***^
Epithelial vacuolation	0.00 ± 0.00	1.39 ± 0.35^***^	0.64 ± 0.4^***^	2.80 ± 25^***^
hypercellularity	0.00 ± 0.00	1.09^*^ ± 0.41^*^	0.20 ± 0.07	2.18 ± 0.30^***^
*Medullae alteration*				
Haemorrhage/(extravasated RBCs)	0.00 ± 0.00	0.09 ± 0.10	0.20 ± 0.28	1.93 ± 0.39^***^

Data: mean ± SE., 0 = no lesion; 1 = mild effects; 2 = moderate effects; 3 = severe lesions. G1: control, G2: therapeutic design, G3: short-term prophylactic design, G4: long-term prophylactic design. *P* values: No asterisks = non-significant (*P* > 0.05), *Significant (*P* < 0.05), **Highly significant (*P* < 0.01), ***Very highly significant (*P* < 0.001) compared to their control, using one-way ANOVA

The alterations were pronounced with variable degree of severity as shown in G2 and G4. Such alterations became more sever in G2 and G4, while in G3, such alterations were restricted to limited spots in the examined sections of this group. The kidney sections showed marked necrosis of the tubules. In the cortices, the glomeruli displayed large mesangial spaces and disturbed capillary architecture. The glomeruli were either clefted, shrunken or degenerated. The epithelium of the proximal and distal tubules displayed variable degrees of stainability. Some of these epithelial cells appeared faintly stained, while other cells showed homogeneous dark stain with pyknosis and/karyolysis. Mainly, most of the necrotic cells fell into the tubular lumen obliterated them. In various sections, the intertubular spaces were widened and this was accompanied with inflammatory infiltrate. The interstitial areas among the renal tubules together with the lumina of the renal tubules were congested with blood cells, which were highly obvious in Fig. 5E–G. The aforementioned figures exhibited widened, thickened and damaged renal blood vessels.

In the medullary region, the examined sections of G4 showed marked necrotic degeneration of the epithelial renal tubules accompanied with mononuclear cells infiltration, as shown in Fig. 6B, compared to that of the control section (Fig. 6A). Also, this was accompanied with several spots of tubular congestion and haemorrhage (such alteration was markedly pronounced and restricted to this group. The degree of histopathological alterations indices in the kidneys of each group due to OP administration were semi-quantitatively analysed, summarized and represented in Table 1 compared to the control group.

### Biochemical findings

The serum markers of hepatic/renal toxicities (ALT, ALP, GGT, direct bilirubin, urea, creatinine, uric acid, Na^+^ levels) were significantly increased (*P* < 0.05–0.001) in rats of G2 (treated with Tamiflu twice daily for 5 days) and in rats of G4 (treated with Tamiflu once daily for 45 days) compared to the control group (G1); as shown in Table 2. On the other hand, no significant changes recorded (*P* > 0.05) in total, and direct bilirubin levels in the rats of either G2 or G4 compared to G1. Moreover, levels of serum total protein, albumin and K^+^ were significantly decreased (*P* < 0.05–0.001) in the rats of either G2 or G4 compared to G1. On the contrary, all of the following parameters assessed in this study did not change significantly (*P* > 0.05) in rats of G3 treated with Tamiflu once daily for 10 days except serum albumin was significantly decreased (*P* < 0.05) in rats of G3 compared with the G1.

**Table 2:** The influence of Tamiflu on the serum markers of hepatic/renal toxicities of the various treated groups compared to that of the control group

Parameters			Groups			
			G1	G2	G3	G4
ALT		(IU/L) activity	93.33 ± 9.94	136.70 ± 9064**	118.00 ± 5.57	161.50 ± 4.46***
AST			184.80 ± 23.05	281.00 ± 19.77*	251.50 ± 21.14	301.00 ± 13.47**
ALP			51.19 ± 4.36	69.50 ± 2.39**	61.42 ± 2.05	91.92 ± 2.77***
GGT			3.83 ± 0.30	4.33 ± 0.33**	2.83 ± 0.30	5.50 ± 0.22***
Total	Bili	(mg/dl)	0.57 ± 0.04	0.62 ± 0.07	0.55 ± 0.05	0.70 ± 0.03
Direct			0.27 ± 0.02	0.15 ± 0.03	0.18 ± 0.04	0.15 ± 0.04
Indirect			0.30 ± 0.06	0.40 ± 0.05*	0.37 ± 0.05	0.55 ± 0.02**
Urea			15.67 ± 0.80	19.74 ± 0.89*	17.83 ± 0.54	36.85 ± 1.50***
Creatinine			0.60 ± 0.02	0.68 ± 0.02*	0.64 ± 0.02	0.72 ± 0.02**
Uric acid			1.98 ± 0.02	2.19 ± 0.04**	2.05 ± 0.04	2.92 ± 0.03***
Total protein		(g/dl)	6.42 ± 0.17	5.47 ± 0.32*	5.70 ± 0.16	5.05 ± 0.23**
Albumin			3.36 ± 0.13	2.37 ± 0.20*	2.57 ± 0.20*	2.26 ± 0.19**
Na^+^		(mmol/L)	120.60 ± 0.87	126.70 ± 1.69*	122.50 ± 0.97	136.20 ± 1.82***
K^+^			4.73 ± 0.24	3.79 ± 0.09*	4.38 ± 0.25	3.25 ± 0.14***

Values: mean ± SE. ALT = Alanine aminotransferase, AST = Aspartate aminotransferase, ALP = Alkaline phosphatase, GGT (γ-GT) = Gamma-glutamyltransferase, Total Bili = total bilirubin, Direct Bili. = direct bilirubin, Ind. Bili. = indirect bilirubin. G1: control, G2: therapeutic design, G3: short-term prophylactic design, G4: long-term prophylactic design. *P* values: No asterisks = non-significant (*P* > 0.05), * Significant (*P* < 0.05), ** Highly significant (*P* < 0.01), *** Very highly significant (*P* < 0.001) compared to their control, using one-way ANOVA

### DNA electrophoretic findings

**Fig. 7:**
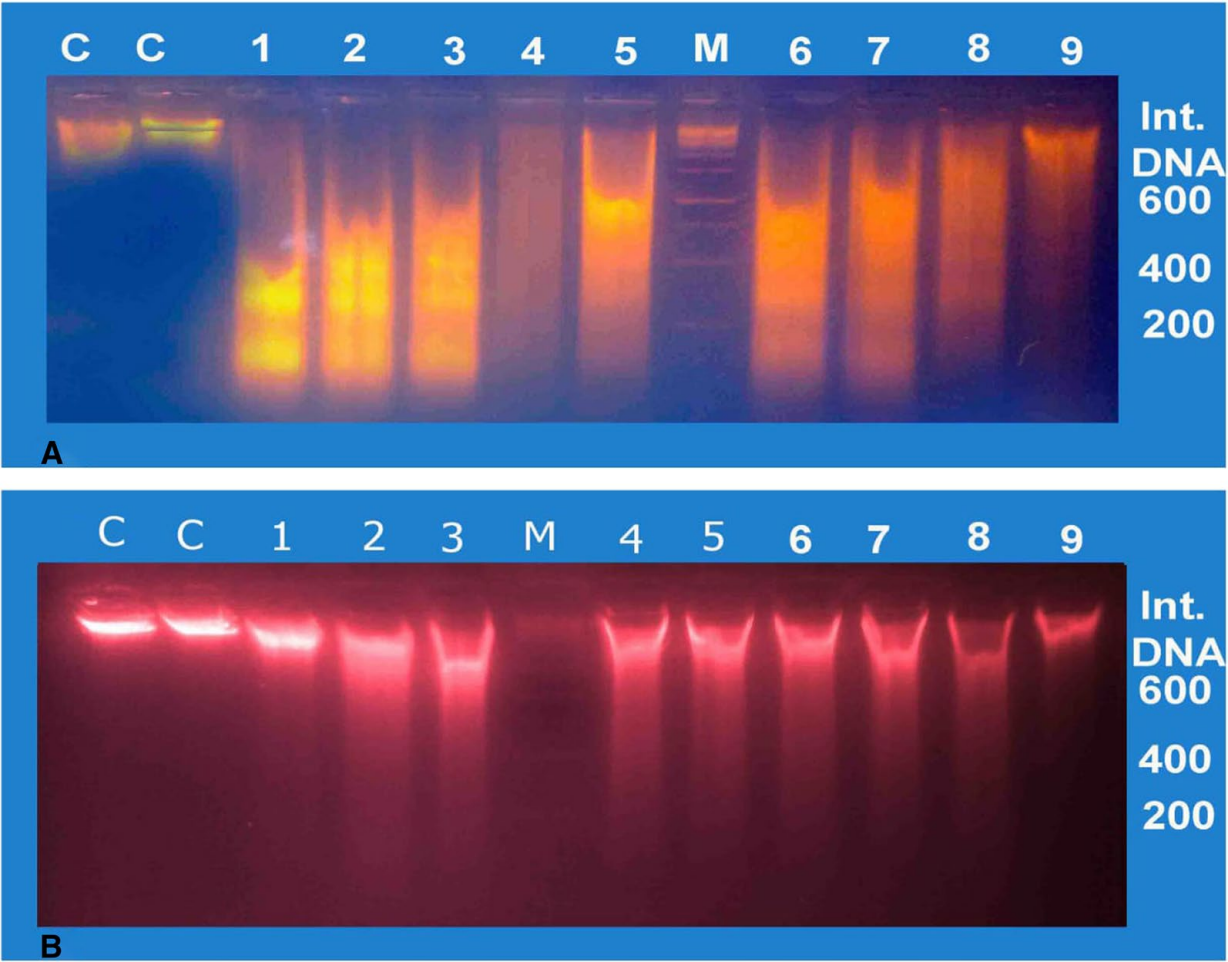
Gel DNA electrophoresis of the liver (**A**) and kidneys (**B**) rats’ specimens. Lanes C, Lanes 1–3, Lanes 4–6, Lanes 7–9: electrophoretic analyses of **G1**, **G2**, **G3** & **G4**; respectively. Lane M = marker (100 bp/DNA ladder). G1: control, G2: therapeutic design, G3: short-term prophylactic design, G4: long-term prophylactic design

The severity of hepatic DNA fragmentation was remarkably increased (P < 0.001) in G2 at 600, 400 and 200 bp that match the mean values 138.90 ± 7.14, 141.70 ± 5.04 and 138.70 ± 3.87, respectively; as well as in G4, their mean values equal 154.80 ± 3.36, 181.2 ± 3.12 and 162.90 ± 4.10, respectively; compared with that of the control group represented by 76.40 ± 5.18, 46.21 ± 5.13 and 27.15 ± 2.92, respectively; as shown in Fig. 7A (lanes 4, 5 & 6) and Table 3. In addition, a very highly significant increase of the hepatic DNA optical density (*P* < 0.001) was obtained in the mean values at 600 and 400 bp equal 117.70 ± 8.40 and 102.20 ± 3.62, respectively, in G3 compared with the obtained mean values of the control group that equal 76.40 ± 5.10 and 46.21 ± 5.13, respectively, as shown in Figs. 7A (lanes 7, 8 & 9) and Table 3. On the other hand, the mean value of the hepatic DNA optical density at 200 bp location of G3 (29.34 ± 2.12) did not show any change (*P* > 0.05) compared to the control group (27.15 ± 2.92).

**Table 3:** The mean values of the optical density of necrotic smears of DNA at 200/400/&600 bp locations and the intact DNA of the liver and kidneys cells of rats treated with Tamiflu compared to G1

Groups	DNA Ladder in bp			
	Intact DNA	600 bp	400 bp	200 bp
**G1**				
Hepatic	187.40 ± 2.40	76.40 ± 5.18	46.21 ± 5.13	27.15 ± 2.92
Renal	192.70 ± 5.24	34.66 ± 5.68	25.83 ± 7.93	23.67 ± 5.69
**G2**				
Hepatic	91.12 ± 9.41***	138.90 ± 7.14***	141.70 ± 5.04 ***	138.70 ± 3.89***
Renal	122.30 ± 7.014***	116.3 ± 12.204***	54.67 ± 11.80	58.00 ± 4.804**
**G3**				
Hepatic	71.92 ± 11.50***	117.7 ± 8.400***	102.20 ± 3.62***	29.34 ± 2.12
Renal	169.00 ± 9.42	43.73 ± 5.60	34.00 ± 8.12	22.33 ± 7.89
**G4**				
Hepatic	143.6 ± 2.54**	154.80 ± 3.36***	181.2 ± 3.12***	162.90 ± 4.10***
Renal	57.41 ± 8.984***	142.00 ± 4.044***	124.10 ± 7.764***	65.83 ± 6.254***

Values: means ± SE. one-way ANOVA. G1: control, G2: therapeutic design, G3: short-term prophylactic design, G4: long-term prophylactic design. *P* values: No asterisks = non-significant (*P* > 0.05), *Significant (*P* < 0.05), **Highly significant (*P* < 0.01), ***Very highly significant (*P* < 0.001) compared to their control, using one-way ANOVA

In Fig. 7B and Table 3, significant increase (*P* < 0.01, and *P* < 0.001) were shown in the mean values of the kidneys’ DNA optical densities  at 200 and 600 bp locations of G2 equal 58.00 ± 4.80 and 122.30 ± 7.01, respectively; also, a significant increase (*P* < 0.001) were observed in the mean values of the kidneys’ DNA optical densities at 200, 400 and 600 bp locations of G4 which recorded as: 154.80 ± 3.36, 124.10 ± 7.76 and 142.00 ± 4.04, respectively; compared with that of the control group: 23.67 ± 5.69, 25.83 ± 7.93 and 34.66 ± 5.68, respectively. On the other hand, the mean values of the kidneys’ DNA optical densities at 200, 400, 600 bp location of G3 did not show any remarkable alterations (*P* > 0.05) compared to the control group.

## Discussion

It is worth to mention that till now there are no data on the safety, tolerability, or even effectiveness of the influenza vaccines in the patients with mild COVID-19 or even with those who have recovered from COVID-19. Therefore, the safety and efficacy of vaccinating persons have not been documented yet [23]. So, this study was carried out to investigate the probable effect of Tamiflu which listed as the main drug in the treatment protocol.

Our biochemical findings showed significant increases in the serum toxicity markers of both liver and kidneys (ALT, AST, ALP, GGT, indirect bilirubin, urea, creatinine, uric acid and Na^+^), which were accompanied by decreases in the levels of serum total protein, albumin and K^+^ ions of G2 (The therapeutic dose) and G4 (long-term prophylactic dose). On the other hand, neither the liver’s nor kidneys’ serum functions were affected by Tamiflu in G3 (short-term prophylactic dose); except the serum albumin level which was significantly decreased compared with the control group. Al-Rikabi [10] and [11] reported significant increases of hepatic and renal serum markers in the rats that treated with Tamiflu (1 mg/kg/6 weeks), with significant increase in serum ALP, direct bilirubin and uric acid, and significant decrease in ALT activity and indirect bilirubin. Also, the results by El-Sayed and Al-Kahtani [24] showed significant elevations in AST and ALT activities in male and female rats that dosed with Tamiflu (2.2 mg/kg/5 days); the same dose caused oxidative stress and acute toxicity in both genders. Akanji et al*.* [25] reported that the increase in the serum enzymes as ALT, AST, ALP, GGT might be due to permeability alterations in the plasma membrane leading to enzyme leakage from liver tissues into the blood stream, leads to hepatocellular functions impairment (such as pyknosis/necrosis).

Significant increases in the liver enzymes markers in G2 and G4 imply a loss of cellular membrane integrity. Aminotransferases catalyse the interconversion of AAs and oxoacids by transferring amino groups in protein metabolism and gluconeogenesis. ALP is a potent anti-inflammatory mediator, protects tissues from damage/injury. Also, it plays an integral role in metabolism and biosynthesis of energy macromolecules within the liver, and breaks down proteins and catalyses the removing of phosphoric esters. AST, ALT and GGT are the most important indicators of hepatocellular injury/necrosis, inflammation and metabolic disorders [26].

The main cause of hyperbilirubinemia could be due to rise an indirect serum bilirubin (unconjugated bilirubin), which may result from poor conjugation, or decreased liver uptake of an indirect bilirubin or/and rapid synthesis (haemolysis) in drug induced liver injury [27]. Moreover, elevation of serum indirect bilirubin in G2 and G4 may be due to free fatty acids which displace indirect bilirubin from its attachment to plasma albumin [28]. Hypoproteinemia can also be caused by liver impairment, which reduces the synthesis of plasma proteins like albumin (hypoalbuminemia) [27]. Other previous studies indicated that the protein depletion referred to impairment in the secretory function of the liver arising probably from hepatocellular injury [29], or increased catabolism [30]. Hyperlipidemia is a well-known risk factor for fatty liver infiltration, which can lead to liver failure. Hypercholesterolemia has been linked to an increase in the production of oxygen free radicals, which has been linked to negative effects on organ tissues such as blood vessels, liver and kidney [27]. All of the aforementioned alterations represent either an increase or a decrease in a certain parameter(s) investigated in this study.

Xenobiotics caused oxidative stress, which is shown histologically. The results of the present study showed foamy/cytoplasmic vacuolization. The latter alteration was explained by Santucci et al*.* [31] due to accumulations of the neutral lipids. The latter cause the foamy appearance of the cells, originate at the ER, accumulate in it and pinched off the ER membrane into the cytosol. Abdel-Ghaffar [13] mentioned that the accumulation of the lipid droplets was a prominent alteration observed ultrastructurally in transverse sections of the testes of Tamiflu-administrated rats.

Also, mononucleated cells infiltration (Küpffer cells/LGC/histiocytes) and haemorrhage (dilatation of PoV/congested BS) were seen in the present study which indicate chronic inflammation. The previous observations agreed with those observed by Michael and Cynthia [32]**.** The authors explained that mononucleated and Küpffer cells activation and others promoting due to tissue damage. While Farrag et al*.* [33] explained that the drug caused the changes should be listed among “drugs-induced liver injury”. The latter type of drugs defined as “drug-induced critical alterations of hepatocytes that trigger the immune systems of susceptible hosts to infiltrate their livers and assault/damage hepatocytes, which leads to liver injury”. Kupffer cells, which are found in the normal liver, play an important role in both hepatic immunological homeostasis and the immunopathology of liver disorders. In response to acute/chronic inflammation, Kupffer cells generate high levels of proinflammatory cytokines and activation of these cells is a critical factor of peroxynitrite production and the severity of liver damage [34]. Moreover, the creation of neoantigens by medicines or their metabolites interacting with host proteins, such as albumin, could be the starting point for initiation of other immune cells, particularly cytotoxic T cells, resulting in liver injury. Hepatic toxicity caused by drugs might be dose/time-dependent or caused by a drug immune response mechanism [35]. In the present study, the liver injury attributable to Tamiflu shows that immuno-allergic factors, resulting from high dose or long duration of treatment, may have caused the liver damage. On the same manner, a previous study reported that OP had a hepatotoxic impact in rats given orally a therapeutic dose of one mg/kg.BW for various periods [10].

Also, as seen in G2 and G4, the nuclear alterations (pyknosis/karyorrhexis/karyolysis) in hepatocytes may be attributed to interference with DNA and protein synthesis in response to Tamiflu-toxic effects.

Hepatic fibrosis was shown in the branches of the hepatic portal veins and the central veins that exhibited markedly thickened endothelial walls. According to Amin et al*.* [36]**,** fibrosis initiated by parenchymal cell destruction due to multiple injurious agents, and followed by inflammation, the latter activates resting hepatic stellate cells.

Tissue injury might result in a high plasma urea level. A high uric acid level is most commonly caused by inefficient uric acid elimination by the kidneys. Urate crystals can form when uric acid builds up, causing kidney injury [37]. So, the elevations in the levels of serum renal toxicity markers (such urea and uric acid) at G2 and G4 might be attributed to impairment/obstruction of protein and nucleic acid catabolism (an indication of the nephrotoxicity of Tamiflu), as it adversely affected the tubular and glomerular function of the rats [11]. On the other hand, creatinine is not reabsorbed; instead, it rises as a result of a lower glomerular filtration rate, which is used to detect renal impairment [37]. When the kidneys fail, the balance of fluid and electrolytes is disrupted, resulting in an imbalance of specific electrolytes. Hypokalemia can also affect the kidneys' ability to concentrate urine, leading to excessive urination and thirst (polyuria and polydipsia, respectively). Also, it can cause several alterations in kidney function, including impaired tubular transport and the development of chronic tubulointerstitial dysfunction [38]. The ability of the kidneys to maintain water homeostasis is known to be affected by chronic renal disease, and thus the risk of both hypo- and hypernatremia, with water changes resulting to cellular swelling or shrinking, can increase as the disease progresses [39]. In this study, the elevated serum creatinine concentration and electrolytes such as Na^+^, as well as the reduction in concentration of serum K^+^ indicate tubular dysfunction [30]. Kang et al*.* [40] concluded that hypernatremia does occur when there is loss of body fluids containing less Na^+^ than plasma. Nduka [41] reported that Na^+^ increase is suspected to be due to the inability of the kidneys to excrete adequate Na^+^ ion from the tubular fluid. These might explain the excess of Na^+^ ion levels in the Tamiflu-administrated rats (G2&4). Earlier researches [11, 42, 43] reported that renal impairment in the highest-dose group (761 mg/kg) of Tamiflu was accompanied by increased water intake, increased leukocyte count and increased bilirubin, urea, creatinine, and urine volume. Also, the same researchers found that the renal histological examination of their studies revealed degenerating/regenerating changes in the renal tubular epithelia, basement membranes and Bowman capsules; vacuolization in the renal tubular epithelia; and mineralization of tubules of renal medulla, as seen in our study. Glomerular dilation, degeneration of the epithelial cells lining the renal tubules, infiltration of inflammatory cells, hyperemia of medullary and cortical parts infiltrates were evident especially in G2 and G4 treated with Tamiflu. In previous studies in rats, experimental hyperuricemia, hyperuricemia and hypercreatininemia have also been associated with the development of mild renal disease, characterized by mild proteinuria, changes in renal blood vessels, glomerular damage, tubulointerstitial fibrosis, stimulation of inflammatory mediators and glomerulosclerosis [40, 44].

We know that OP is hydrolysed by hepatic carboxylesterases to oseltamivir carboxylate (its active metabolite), that excreted by the kidneys through glomerular filtration and renal tubular secretion [3]. Farrely [45] reported that repetitive doses/long-term use of Tamiflu caused the kidneys to become unable to hydrolyse the oseltamivir/Tamiflu sufficiently. So, its active metabolite forms excessive quantities of ph^−^ will accumulate, leading to mineralization of the kidneys. This may explain the obtained results of this work. According to Basile et al*.* [46], the damage of the four major structures of the kidney (the tubules; the glomeruli; the interstitium; and the intrarenal blood vessels) is the major reason resulted in acute kidney injury.

In addition, the biochemical and histopathological alterations observed in the present study were in parallel with the molecular findings. The production of reactive oxygen species (ROS) by Tamiflu toxic metabolism or the consequent mitochondrial damage might cause direct or indirect oxidative DNA damage. Our results of hepatic DNA electrophoresis demonstrated that the oral administration of Tamiflu significantly induced necrosis, especially in G2 and G4 (therapeutic and long-term prophylactic doses) at three bp location (600, 400 and 200 bp), which increased gradually depending on the doses accumulation (dose-dependent) as well as long duration (time-dependent). While the integrity of hepatic DNA in G3 (short-term prophylactic dose) gave necrotic hepatic DNA smears at two bp location (600, and 400 bp) only. In addition, renal DNA integrity in G2 and G4 (the therapeutic and long-term prophylactic doses) gave necrotic DNA smears at two bp location (200 and 600) and three bp location (600, 400 and 200 bp), respectively. On the other hand, renal DNA integrity of G3 at 200, 400 and 600 bp location did not show any significant change compared to the control group. Thus, the effect of the examined drug on the integrity of DNA in the liver samples was higher than in the kidney’s samples. The optical density gradually increased depending on the doses accumulation as well as long duration. El-Ganzuri et al. [12] and Abdel-Ghaffar [13] reported that the therapeutic dose, short-term and  long-term prophylactic doses gave necrotic DNA smears, but the intensity of such dose was both time- and dose-dependent.

Finally, the reasons that lead to hepatoxicity and nephrotoxicity due to Tamiflu administration should be adequately focused and addressed. In general, increased vigilance during pre-clinical drug/vaccines development and clinical trials, serum hepatic/renal enzymes monitoring with particular medications and the potential finding of both diagnostic/prognostic biomarkers are all ways to prevent drug hepato- or/and renal toxicities.

## Conclusions

It was found that Tamiflu/OP at its therapeutic and long-term prophylactic doses caused structural and functional hepato- and nephrotoxicity; hence, it should be consumed with caution and not used for long periods (time-dependent) or/and repetitive doses (accumulative-dose-dependent) especially with patients suffer from liver or/and kidney dysfunction, while the short-term prophylactic dose of Tamiflu appear to be relatively safe and could be explored for oral medications.

## Data Availability

The datasets supporting the conclusions of this article are included within the article.

## Abbreviations

ALT: Alanine aminotransferase
AST: Aspartate aminotransferase
ALP: Alkaline phosphatase
GGT (γ-GT): Gamma-glutamyltransferase
Total Bili: Total bilirubin
Direct Bili.: Direct bilirubin
